# Deep Learning-Based Diagnosis of Alzheimer’s Disease

**DOI:** 10.3390/jpm12050815

**Published:** 2022-05-18

**Authors:** Tausifa Jan Saleem, Syed Rameem Zahra, Fan Wu, Ahmed Alwakeel, Mohammed Alwakeel, Fathe Jeribi, Mohammad Hijji

**Affiliations:** 1 Department of Computer Science and Engineering, National Institute of Technology Srinagar, Srinagar 190006, J&K, India; tausifa_30phd17@nitsri.net (T.J.S.); rameemzahra_36phd17@nitsri.net (S.R.Z.); 2 Department of Computer Science, Tuskegee University, Tuskegee, AL 36088, USA; fwu@tuskegee.edu; 3 Sensor Network and Cellular Systems Research Center, University of Tabuk, Tabuk 71491, Saudi Arabia; 4 Faculty of Computers & Information Technology, University of Tabuk, Tabuk 71491, Saudi Arabia; alwakeel@ut.edu.sa (M.A.); m.hijji@ut.edu.sa (M.H.); 5 College of Computer Science and Information Technology, Jazan University, Jazan 45142, Saudi Arabia; fjeribi@jazanu.edu.sa

**Keywords:** Alzheimer’s disease, deep learning, biomarkers, positron emission tomography, Magnetic Resonance Imaging, mild cognitive impairment

## Abstract

Alzheimer’s disease (AD), the most familiar type of dementia, is a severe concern in modern healthcare. Around 5.5 million people aged 65 and above have AD, and it is the sixth leading cause of mortality in the US. AD is an irreversible, degenerative brain disorder characterized by a loss of cognitive function and has no proven cure. Deep learning techniques have gained popularity in recent years, particularly in the domains of natural language processing and computer vision. Since 2014, these techniques have begun to achieve substantial consideration in AD diagnosis research, and the number of papers published in this arena is rising drastically. Deep learning techniques have been reported to be more accurate for AD diagnosis in comparison to conventional machine learning models. Motivated to explore the potential of deep learning in AD diagnosis, this study reviews the current state-of-the-art in AD diagnosis using deep learning. We summarize the most recent trends and findings using a thorough literature review. The study also explores the different biomarkers and datasets for AD diagnosis. Even though deep learning has shown promise in AD diagnosis, there are still several challenges that need to be addressed.

## 1. Introduction

Alzheimer’s disease (AD) is the most widespread neurodegenerative disease, with a prefatory Mild Cognitive Impairment (MCI) stage in which memory loss is the primary symptom, which gradually worsens with conduct problems and deprived self-care [1]. However, not everyone identified as having an MCI goes on to develop AD [2]. A small percentage of people with MCI develop non-AD dementia or stay stable in the MCI stage without advancing to dementia [2]. Even though there is no cure for AD, it is vital to correctly recognize those in the MCI phase who will develop AD. Simultaneously, it would be ideal to correctly identify people in the MCI stage who do not advance to AD so that they are saved from unneeded pharmacologic therapies that at best may give little help and, at worst, may harm them more with side effects. As a result, much work has gone into developing early detection tools, particularly at pre-symptomatic phases, in an attempt to reduce or thwart disease progression. Advanced neuroimaging strategies, such as Magnetic Resonance Imaging (MRI) and Positron Emission Tomography (PET), have been employed to uncover the structural and molecular biomarkers pertaining to AD [3].

Brisk advancement in neuroimaging strategies has made the integration of large-scale and high-dimensional multi-modal neuroimaging data very crucial [4]. As a result, interest in computer-assisted machine learning methodologies for integrative analysis of neuroimaging data has attracted a lot of attention. Well-known machine learning approaches such as Support Vector Machine (SVM), Linear Discriminant Analysis (LDA), Decision Trees (DT), etc., have been employed and promise early diagnosis and prediction of AD progression. However, appropriate pre-processing steps must be applied before using such approaches. Moreover, these approaches require feature extraction, feature selection, dimensionality reduction, and feature-based classification for classification and prediction. These steps necessitate specialist knowledge as well as several optimization stages, which are time-intensive [5]. To overcome these hurdles, deep learning (DL), a looming domain of machine learning research that employs raw neuroimaging data to produce features through “on-the-fly” learning, is garnering substantial attention in the field of large-scale, high-dimensional neuroimaging analysis.

Motivated to unfurl the power of DL techniques in AD diagnosis, we present an extensive review of the current state-of-the-art in the area of DL-based AD diagnosis. More precisely, this paper;

- Investigates the biomarkers for AD;
- Explores the different AD datasets;
- Discusses the different DL techniques;
- Reviews the most recent literature pertaining to DL-based AD diagnosis;
- Presents the trends and key findings from the literature review;
- Highlights the obstacles that the scientific community still faces in this area.

The remainder of the paper is organized as follows: Section 2 presents the preliminaries required for understanding the DL-based AD diagnosis. These preliminaries are crucial for understanding the most suitable biomarkers for AD diagnosis, the AD datasets and the appropriate DL techniques for those datasets and how DL is used for AD diagnosis. Section 3 reviews the literature pertaining to DL-based AD diagnosis. The literature is classified based on the DL technique used. This section also presents the tabular summary of the reviewed literature highlighting the year of publication of the study, biomarker used, DL technique used, AD dataset used, and the performance achieved by the study. Section 4 discusses the key findings from the literature review. The section also presents a four-panel graph plotting the number of studies versus biomarkers, number of studies versus AD datasets, number of studies versus the DL technique, and number of studies versus performance metrics. Section 5 highlights the challenges faced by DL in AD diagnosis. Finally, Section 6 presents the concluding remarks.

## 2. Preliminaries

### 2.1. Biomarkers for AD

A large subgroup of medical indicators, objective signals of medical state viewed from outside the patient that can be assessed correctly and reproducibly, is referred to as a biomarker. The biomarkers of AD include Magnetic Resonance Imaging, functional Magnetic Resonance Imaging (fMRI), Fluorodeoxyglucose-Positron Emission Tomography (FDG-PET), Amyloid-Positron Emission Tomography (Amyloid-PET), Tau-Positron Emission Tomography (Tau-PET), electroencephalography (EEG), magnetoencephalography (MEG), speech transcripts, genetic measures and cerebrospinal fluid (CSF) measures. Following provides a detailed description of these biomarkers.

#### 2.1.1. MRI

The non-invasive in vivo imaging of the human brain with MRI is a powerful method [6]. It can help to characterize neurological diseases such as AD quantitatively. Even before clinical signs or irreparable brain damage are evident, MRI can give useful biomarkers. Numerous studies have found links between quantitative measurements derived from brain MR images and the course of AD. There is significant evidence that different areas of the brain are impacted at various stages of the disease, with the hippocampus, amygdala, and entorhinal cortex showing early involvement [6]. Although these markers are sensitive to dementia, they may not be adequately specific to AD. A standardized strategy that takes into account pathological alterations in several areas throughout the brain has the potential to improve dementia diagnosis specificity and the differential diagnosis of different forms of dementia. As a result, rather than focusing on a small number of brain structures, it is preferable to take a holistic approach and examine a vast number of structures throughout the brain.

The hippocampus and cerebral cortex shrink, and the ventricles grow, as AD progresses. The severity of these consequences varies according to the stage of the disease. Dramatic shrinkage of the hippocampus and cerebral cortex, and enlargement of ventricles, occurs in the advanced stages of AD. These changes are quite noticeable in the MR images. Automatic approaches such as DL perform in a manner comparable to expert radiologists while classifying MR images of Alzheimer’s patients.

#### 2.1.2. fMRI

Functional brain alterations are anticipated to precede structural brain abnormalities. Hence, resting-state fMRI, a technique used to scan intrinsic functional brain connections, is a prospective diagnostic for AD [7]. Resting-state functional connectivity has been found to be responsive to functional brain alterations associated with AD in previous research.

#### 2.1.3. FDG-PET

In the assessment of individuals with suspected neurodegenerative illnesses, particularly AD, FDG-PET is widely and increasingly utilized to support the clinical diagnosis [8]. It represents synapse loss, and neuronal functional impairment. Lower FDG-PET levels were thought to be a marker of neuronal hypo-metabolism caused by neuro-degeneration. However, it has been found that it reflects the glucose consumption of astrocytes rather than neurons [8]. Furthermore, there is evidence that decreased FDG brain uptake by PET could be a diagnostic for blood–brain barrier (BBB) transport abnormalities [8]. 

#### 2.1.4. Amyloid-PET

Amyloid-PET is a type of neuroimaging that uses standardized visual reading protocols for each tracer. It enables the non-invasive, in vivo identification of amyloid plaques, the key neuro-pathological indicators of AD, with extremely high values of sensitivity and specificity in patients with diagnosed AD who had an autopsy within one year of PET scanning [9]. Amyloid-PET can also detect amyloid pathology in clinically unusual AD variants that include logopenic variants, frontal-executive variants and posterior cortical atrophy [9]. However, it does not allow for the distinction of different amyloid-positive illnesses with comparable amyloid-deposition patterns.

#### 2.1.5. Tau-PET 

The drawbacks of amyloid imaging include the fact that amyloid plaques alone are not enough to diagnose AD. As a result, the development of a PET tau-pathology tracer could be used as a supplement to help with positive diagnosis and staging of AD [10]. Extracellular amyloid-β deposits and neurofibrillary tangles of intracellular incorrectly folded phosphorylated tau (p-tau) protein are neuropathological indicators of AD. In AD, tau protein undergoes chemical alterations (hyper-phosphorylation). It has been observed that when tau threads become hyper-phosphorylated, they tangle together and form paired helical filaments, triggering microtubule disintegration, and they crumple the transport system of neurons and create intractable aggregates. With this result, neuronal communication is disrupted, which leads to cell death. The presence of neurofibrillary tangles shows a hierarchical spreading pattern of tau pathology, in contrast to the diffuse distribution of amyloid plaques in the neocortex [11]. A prudent procedure would be to apply image staging methods for analyzing the location and quantity of tau tracer retention in PET. The contemporary advancements in PET tau tracers facilitate the visualization, mapping, quantification and examination of tau pathology in relation to cognition [12,13,14]. Hence, Tau-PET imaging is a significant advancement in AD diagnosis, and it is hoped that the combination of amyloid positive and tau positive PET scans will get us closer to an affirmative in vivo diagnosis of AD in the future.

#### 2.1.6. EEG

Clinical indicators such as EEG, quantitative EEG, event-related potential, transcranial magnetic stimulation, and vagus nerve stimulation can identify neural alterations linked with dementia [15]. EEG is a neurosignal that accurately measures information processing in milliseconds. It undergoes visual evaluation by clinicians and has yielded adequate diagnosis results. However, EEGs have a lower spatial resolution than other neuroimaging techniques, despite the fact that these techniques do not provide functional information about the brain and have a limited temporal resolution; EEG, on the other hand, has a high temporal resolution and is thus essential for monitoring the brain activity. Hence, the degree of abnormality in EEG can be used to determine the intensity of dementia and AD. 

#### 2.1.7. MEG

While EEG gives valuable information on AD, it does not appear appropriate for detecting preclinical illness [15]. Prior to the onset of symptoms, MEG is utilized to identify functional abnormalities in the brain caused by AD [16]. It is a method for quantifying magnetic fields in the brain that are generated due to electrical currents, and it has been particularly beneficial in non-invasive epilepsy research. It can examine the activations of synchronously firing neuronal populations with a sub-millisecond temporal precision and reports brain activity depending on magnetic fields created by coordinated neuronal currents. MEG usually employs far more detectors than typical EEG sensor arrays, allowing for quick signal coverage [16]. High-frequency bands have higher SNR values than EEG, which is impacted by biological tissue resistance and distorted by the skull, since magnetic fields are more visible through biological tissue. In comparison to EEG, this provides for improved spatial resolution and consequently improved localization of electrical activity sources from MEG. 

#### 2.1.8. Speech Transcripts

Linguistic ability as measured by oral declarations could be an excellent indicator of AD and other dementias [17]. The idea is that neurodegenerative diseases cause nerve cells that manage language, speech and cognitive functionalities to degrade, which affects the way patients create oral statements. 

#### 2.1.9. Genetic Measures

In identifying the mechanisms behind AD, genomic and genetic methods have made significant progress [18]. Twenty-three statistically significant AD genes have been revealed by meta-analyses and Genome-Wide Association Studies (GWAS). So far, thirty-nine AD risk genes have been discovered, including APOE, SORL, TRIP, ABCA, and APP [18]. These genes emphasize the significance of several functionalities implicated in AD, including cell migration, hippocampal synaptic function, immune response and inflammation, lipid transport and endocytosis, and other cell regulatory mechanisms, as well as the impact of tau and amyloid protein. The bulk of the literature available in gene expression has used post-mortem brain samples, and, as a result, they have primarily stressed severe illness phases. 

#### 2.1.10. CSF Measures

CSF is a translucent substance that surrounds the brain and fills the subarachnoid space and ventricular system [6]. It is a “liquid cushion” that protects the brain mechanically and immunologically. It is collected through a lumbar puncture. Despite the fact that lumbar puncture is intrusive and, most likely, uncomfortable for the patient, CSF is the most revealing substance in the search for biomarkers for neurodegenerative diseases [6]. Because CSF is not isolated from the brain by the firmly regulated BBB, it has closer proximity with the brain than any other substance. Hence, proteins or peptides that are direct indicators of brain-specific activities or disease pathology are more likely to disseminate into CSF than any other physiological substance. These metabolites and proteins have the potential to be useful biomarkers for AD and other neurodegenerative illnesses. CSF indicators might be especially useful in the beginning phases of AD, when determining the proper diagnosis is the most challenging. 

Amyloid-β and tau in CSF are the most commonly used biomarkers for AD. Both the proteins are related to amyloid plaques and neurofibrillary tangles, which are two characteristic lesions of AD. Senile plaques, predominantly made up of amyloid-β, a proteolytic fragment of the amyloid precursor protein, are one of the major pathological hallmarks of AD. The degree of amyloid precursor protein expression could be used as a diagnostic sign in AD. Tau is an intracellular protein that maintains the stability of microtubules in neurons. Normal individuals have a very small concentration of tau in CSF. A multitude of post-translational changes, including phosphorylation at threonine and serine residues, affect the functioning of tau. These changes could lead to the loss of axonal integrity and increased cytoskeleton flexibility in the brain [19]. Tau-protein is discharged into CSF when the neuronal architecture gets significantly disrupted. As a result, higher tau levels in CSF mark the onset of neurodegeneration in AD. P-tau in CSF is also being used as a potential biomarker of AD, as it is the major component of neurofibrillary tangles. CSF concentration of p-tau in AD patients has been found to be higher than normal individuals. The increased p-tau concentration discriminates AD from normal aging and other dementia in a more accurate manner than that of CSF concentrations of total-tau (t-tau) and Aβ-42 [20,21,22]. 

### 2.2. AD Datasets

The most extensively used datasets for AD diagnosis include Alzheimer’s Disease Neuroimaging Initiative (ADNI) dataset, Open Access Series of Imaging Studies (OASIS) dataset, DementiaBank, Harvard Aging Brain Study (HABS) dataset, and Mayo Clinic Study of Aging (MCSA) dataset. They are discussed in detail below.

#### 2.2.1. ADNI Dataset

**Figure 1:**
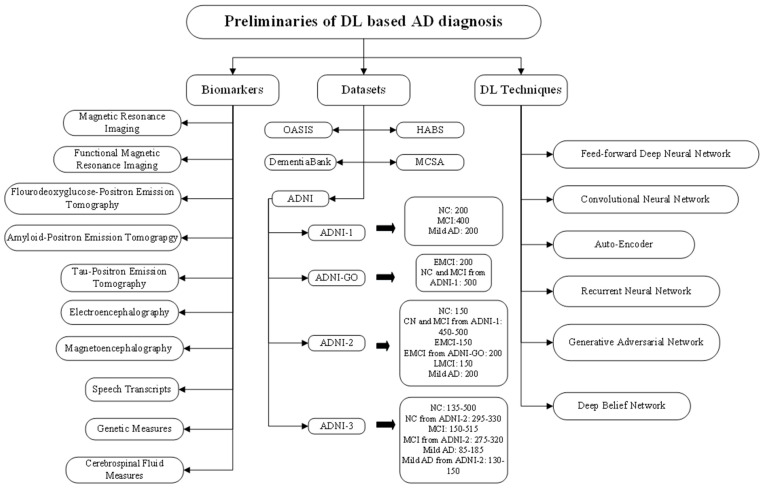
Presents the preliminaries required for DL-based diagnosis of AD. These preliminaries include biomarkers of AD, AD datasets and DL techniques.

The ADNI [23] was introduced in 2003 as a USD 60 million, five-year public-private joint venture by the National Institute of Biomedical Imaging and Bioengineering, the National Institute on Aging, private pharmaceutical organizations, the Food and Drug Administration, and some non-profit groups. ADNI is a longitudinal study and its major purpose is to see if serial MRI, CSF measurements, PET, clinical assessments and other neuropsychological factors could be used to track MCI and early AD progression. The identification of responsive and precise indicators of premature AD diagnosis will benefit clinicians and research scientists in developing novel and effective treatments and keeping track of their efficacy, as well as shortening the time and expense of clinical examinations. Michael W. Weiner, MD, of the VA Medical Center and the University of California, San Francisco, is the initiative’s Principal Investigator. ADNI is the consequence of the collaborative hard work of several co-investigators from a variety of academic institutions and corporate businesses, and subjects have been taken from over 50 locations across the United States and Canada. Depending on the participant pool, ADNI datasets are classified into four types: ADNI-1, ADNI GO, ADNI-2, ADNI-3, as shown in Figure 1.

#### 2.2.2. OASIS Dataset

OASIS [24] is a collection of 1098 participants’ MRI and PET imaging, as well as linked clinical data, gathered over the period of 15 years in the Washington University Knight Alzheimer Disease Research Center. Participants range in age from 42 to 95 years old and include 605 cognitively intact adults as well as 493 people in various phases of cognitive deterioration. About 2000 MR Sessions, comprising numerous functional and structural sequences, are contained in the OASIS dataset. About 1500 raw imaging scans from PET metabolic and amyloid imaging, as well as post-processed images from the PET Unified Pipeline (PUP), are available in OASIS. Post-processed imaging data, such as PET and volumetric segmentation studies, are also accessible in OASIS. APOE and dementia status, as well as longitudinal cognitive and clinical results, are all included in the imaging data. The scientific community can use OASIS as a free-access dataset to answer issues about healthy aging and dementia. Apart from longitudinal dataset, OASIS also consists of a cross-sectional dataset.

#### 2.2.3. DementiaBank Dataset

DementiaBank [25] contains recorded speech of 117 subjects diagnosed with AD and 93 healthy subjects. The subjects are reading an image’s description. The data came from a National Institute on Aging funded longitudinal study on AD and associated dementia done by the University of Pittsburgh, School of Medicine. Speech transcripts of individuals with potential AD, MCI, and other dementias are included in the dataset. Participants answered questions in English depending on the explanation of the Cookie-Theft image, part of the Boston Diagnostic Aphasia Examination (BDAE). The BDAE Cookie-Theft image has been demonstrated to be clinically useful in detecting deficits in linguistic utterances in people with AD and aphasia.

#### 2.2.4. HABS Dataset

HABS [26] is a long-term observational study aimed at better understanding brain aging and the early stages of AD. This research used imaging data to detect early signs of AD, such as tau tangles and amyloid plaques, as well as structural and functional imaging data and thorough assessments of memory and other cognitive functions. The main purpose is to provide information that will speed up progress toward successful prevention of cognitive deterioration caused by AD. The current version of the HABS public dataset (v2.0) contains 290 participants who have been followed for up to 5 years. At the start of the study, the participants’ ages ranged from 62 to 90 years old, and they were all classified as non-clinically impaired. Clinical and cognitive tests are usually taken once a year, while imaging measures are taken at baseline, three years, and five years.

#### 2.2.5. MCSA Dataset

MCSA [27] is a population-based cohort study that aims to find out how common MCI and dementia are, as well as their causes and risk factors. The Rochester Epidemiology Project was used to count the population of Olmsted County, Minnesota, aged 70–89 years old on 1 October 2004. Eligible subjects were chosen at random and invited to take part in the study. A thorough in-person neuropsychological and neurological examination of the participants was carried out. Using previously published criteria, a panel made a consensus diagnosis of NC, MCI, and dementia. A subset of participants was interviewed over the phone. A total of 2719 individuals were assessed, which included 703 women (70–79 years of age), 769 women (80–89 years of age), 730 men (70–79 years of age) and 517 men (80–89 years of age). 2050 participants were assessed in person, while 669 were assessed over the phone. A thorough assessment of the medical data discovered 402 dementia patients.

### 2.3. Deep Learning Techniques

**Figure 2:**
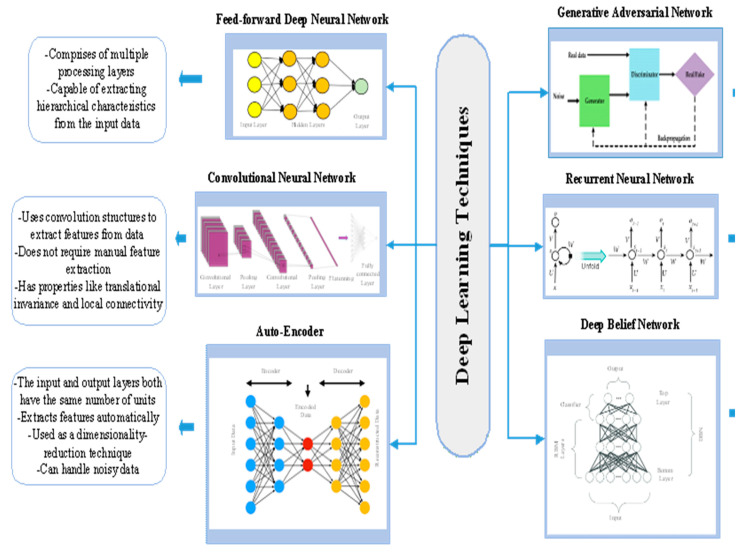
Deep learning techniques.

This sub-section presents a discussion on deep learning techniques. These techniques include feed-forward Deep Neural Network, Convolutional Neural Network (CNN), Auto-Encoder (AE), Recurrent Neural Network (RNN), Deep Belief Network (DBN), and Generative Adversarial Network (GAN). Figure 2 presents their architectures and outlines some of their crucial features.

#### 2.3.1. Feed-Forward DNN

A feed-forward DNN is a collection of neurons arranged in a series of layers, with each layer’s neurons receiving input from the preceding layer and performing a weighted sum of the input followed by a nonlinear activation [28]. A sophisticated non-linear mapping from the input to the output is implemented by the network’s neurons in concert. The weights of each neuron are tuned using a method called error back-propagation to discover this mapping from the data.

#### 2.3.2. CNN

A CNN [29,30] is a feed-forward neural network that uses convolution structures to extract features from data. Unlike classic feature extraction approaches, CNN does not require manual feature extraction. CNN kernels signify diverse receptors that can react to various characteristics, whereas activation functions replicate the function in which only electric signals from neurons over a particular threshold can be sent to the subsequent neuron. CNN has dominance over generic artificial neural networks in the following ways: (1) Local connections: Neurons of a layer are not linked to all neurons from the preceding layer, but only to a limited fraction of them, which helps to reduce parameters and accelerate convergence; (2) Weight sharing: The weights of a collection of links can be shared, thus reducing parameters; (3) Down-sampling dimensionality reduction: To down-sample an image, a pooling layer uses the notion of image local correlation, which reduces the amount of data while preserving important information. It can help cut down on the number of parameters by deleting unnecessary ones. CNN has become one of the most representative algorithms in DL due to its enticing properties.

Convolution is an important phase in the feature extraction process. Convolutional outputs are known as feature maps. Information in the border can be lost while using a convolution kernel of a given size. As a result, padding is used to extend the input with a zero value, allowing the size to be adjusted indirectly. Furthermore, stride is used to adjust the density of convolving. The density decreases with increase in the size of stride. Following convolution, feature maps include a significant number of features, which might lead to an over-fitting problem. As a result, pooling, including max pooling and average pooling, is advocated to eliminate redundancy.

#### 2.3.3. AE

AE [28,29,30] is a typical unsupervised DL algorithm. It is the most common sort of generative model that uses simple neural networks to compress high-dimensional input into a compact representation. The goal of AE is to make the target values identical to the original input. It usually consists of three distinct phases:

- Encoder: The weight matrix and bias are used to parameterize the encoder, which is a series of linear feed-forward filters (analogous to a multi-layer perceptron).
- Activation: The encoded coefficients are transformed into the range [0,1] through activation, a non-linear mapping.
- The input is reconstructed using a collection of reverse linear filters called a decoder.

AE is a standard feed-forward neural network. A neuron’s output can be used as an input by another neuron. A back-propagation algorithm can be used to estimate the parameters. The activations throughout the network, including the hypothesis’ output value, are computed first in a “forward pass”. An “error term” is computed for each middle node, indicating how much that node was liable for any errors in the output. The difference between the network’s activation and the true target value can be assessed directly for an output node. It can be further used to update the “error term”.

#### 2.3.4. RNN

Since the ordinary feed-forward DNN was not capable of modeling the time-series tasks, a modified version of the feed-forward DNN called RNN [28,29,30] was developed to analyze such tasks. The RNN’s input at a specific time ‘p’ is input at time ‘p’ and output at time ‘p−1’. Its neurons are equipped with an internal memory that records previous calculations. Back Propagation Through Time (BPTT), a form of back propagation, is used to train the RNN instead of back propagation.

#### 2.3.5. DBN

DBN [28,29,30] is a form of DNN that is generative. It is made up of numerous hidden layers and a visible layer. DBN extracts complex abstractions from raw data. DBNs are made up of several stacked RBMs. They are trained in an unsupervised manner, with the network learning to probabilistically reconstruct the inputs from the features retrieved at every layer. Apart from generative feature discovery, DBNs can be applied to discriminative prediction problems.

#### 2.3.6. GAN

GANs [28,29,30] are hybrid DL models made up of two components: the discriminator and the generator. These components work together to generate high-quality data. The discriminative network learns to minimize the difference between the original data and the data produced by the generator, whereas the generator learns the data distribution and generates new data based on the acquired data patterns. GANs are ideal for circumstances involving noisy data.

## 3. DL for AD Diagnosis

**Figure 3:**
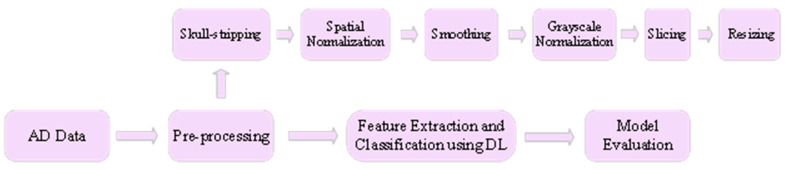
DL-based AD classification framework.

Figure 3 presents a framework for classification of the AD using DL. The AD dataset is pre-processed first using pre-processing techniques such as skull stripping, spatial normalization, smoothing, grayscale normalization, slicing and resizing. Skull stripping is used to segregate non-brain tissues from brain tissues. Spatial normalization normalizes images from diverse subjects to a common template. Smoothing improves the quality of the images by removing noise from the images. Grayscale normalization maps the pixel intensity values to a new and more suitable range. Slicing divides the image into multiple logical images. Finally, resizing is carried out in order to get the desired image size. Then the pre-processed data are fed as input to the DL model that performs feature extraction and classification of the input data. Finally, the model is evaluated using performance metrics such as accuracy, F1 score, area under curve (AUC), and mean squared error (MSE). The following presents a thorough literature review of DL techniques for AD diagnosis. Table 1 presents a summary of these research works.

### 3.1. Feed-Forward DNN for AD Diagnosis

Feed-forward DNN has been utilized by multiple studies for AD diagnosis. Amoroso et al. [31] proposed a method based on Random Forest (RF) and DNN for revealing the onset of Alzheimer’s in subjects with MCI. RF was used for feature selection, and DNN performed the classification of input. The RF consisted of 500 trees and performed 100 rounds, and in each round, 20 crucial features were chosen. DNN consisted of 11 layers with 2056 input units and four output units. ReLU and tanh were used as the activation functions, and categorical cross-entropy was used as the loss function. Adam was used as the optimizer in the DNN. The authors compared the proposed approach with SVM and RF, and it was shown that the proposed method outperforms these techniques.

Kim and Kim [32] proposed a DNN-based model for the diagnosis of Alzheimer’s in its early stage. The model takes the EEG of the subjects as input and classifies it into two groups, MCI and HC (healthy controls). The authors compared the proposed approach with a shallow neural network, and it was demonstrated that the proposed model outperforms a shallow neural network. Rouzannezhad et al. [33] formulated a technique based on DNN for binary (MCI, CN) and multiclass (EMCI, LMCI, AD, CN) classification of subjects in order to detect AD in the premature stage. The authors fed multimodal data (MRI, PET and typical neurophysiological parameters) as input to the DNN. The DNN consisted of three hidden layers, and Adam was used as the optimizer. Moreover, dropout was used to avoid the over-fitting problem. Experiments carried out in the research work demonstrated that the proposed technique performs better than the single modal scenarios in which only MRI or PET was fed as input to the DNN model. Moreover, the fusion of typical neurophysiological data with MRI and PET further enhanced the efficiency of the approach.

Fruehwirt et al. [34] formulated a model based on Bayesian DNN that predicts the severity of AD disease using EEG data. The proposed model consisted of two layers with 100 units each. The authors demonstrated that the proposed model is a good fit for predicting disease severity in clinical neuroscience. Orimaye et al. [35] proposed a hybrid model consisting of DNN and deep language models (D2NNLM) to predict AD. Experiments conducted in the study demonstrated that the proposed model predicts the conversion of MCI to AD with high accuracy. Ning et al. [36] formulated a neural network-based model for the classification of subjects into AD and CN categories. Moreover, the model predicts the conversion of MCI subjects to AD. MRI and genetic data were fed as input to the model. The authors compared the proposed model with logistic regression (LR), and it was demonstrated that the proposed model outperforms the LR model.

Park et al. [37] proposed a model based on DNN that takes as input the integrated gene expressions and DNA methylation data and predicts the progression of AD. The authors demonstrated that the integrated data results in better model accuracy as compared to single-modal data. Moreover, the proposed model outperformed existing machine learning models. The authors used the Bayesian method to choose optimal parameters for the model. It was shown that a DNN with eight hidden layers, 306 nodes in each layer, the learning rate of 0.02, and a dropout rate of 0.85 attains the best performance. Benyoussef et al. [38] proposed a hybrid model consisting of KNN (K-Nearest Neighbor) and DNN for the classification of subjects into No-Dementia (ND), MCI and AD based on MRI data. In the proposed model, KNN assisted DNN in discriminating subjects that are easily diagnosable from hard to diagnose subjects. The DNN consisted of two hidden layers with 100 nodes each. Experimental results demonstrated that the proposed model successfully classified the different AD stages.

Manzak et al. [39] formulated a model based on DNN for the detection of AD in the early stage. RF was used for feature extraction in the proposed model. Albright [40] predicted the progression of AD using DNN in both cases, i.e., the subjects who were CN initially and later got AD and subjects who were having MCI and converted to AD. Suresha and Parthasarathy [41] proposed a model based on DNN with the rectified Adam optimizer for the detection of AD. The authors utilized the Histogram of Oriented Gradients (HOG) to extract crucial features from the MRI scans. It was shown with the help of experiments that the proposed model outperformed the existing strategies by a good margin. Wang et al. [42] utilized gene expression data for studying the molecular changes caused due to AD. The study used a DNN model for identifying the crucial molecular networks that are responsible for AD detection.

### 3.2. CNN for AD Diagnosis

The following studies utilized CNN for AD diagnosis. Suk and Shen [43] proposed a hybrid model based on Sparse Regression Networks and CNN for AD diagnosis. The model employed multiple Sparse Regression Networks for generating multiple target-level representations. These target-level representations were then integrated by CNN that optimally identified the output label. Billones et al. [44] altered the 16-layered VGGNet for classifying the subjects into three categories, AD, MCI and HC, based on structural MRI scans. Experiments conducted in the study demonstrated that the authors successfully performed classifications with good accuracy. The authors claimed that this was achieved without performing segmentation of the MR images.

Sarraf and Tofighi [45] utilized LeNet architecture for the classification of the AD subjects from healthy ones based on functional MRI. The authors concluded that due to the shift-invariant and scale-invariant properties, CNN has got a massive scope in medical imaging. In another study, Sarraf and Tofighi [46] utilized LeNet architecture for classification of AD subjects from healthy ones based on structural MR images. The study attained an accuracy of 98.84%. In one more study, Sarraf and Tofighi [47] utilized LeNet and GoogleNet architectures for AD diagnosis based on Functional as well as structural MR images. Experiments conducted in the study demonstrated that these architectures performed better than state-of-the-art AD diagnosis techniques.

Gunawardena et al. [48] formulated a method based on CNN for the diagnosis of AD in its early stage using structural MRI. The study compared the performance of the proposed method with SVM, and it was shown that the CNN model outperformed the SVM. The authors intend to incorporate two more MRI views (axial view and sagittal view) in addition to the coronal view used in this study in future. Basaia et al. [49] developed a model based on CNN for the diagnosis of AD using structural MR images. The study implemented data augmentation and transfer learning techniques for avoiding the over-fitting problem and improving the computational efficiency of the model. The authors claimed that the study overcomes limitations of the existing studies that usually focused on single-center datasets, which limits their usage.

Wang et al. [50] designed an eight-layered CNN model for AD diagnosis. The authors compared three different activation functions, namely rectified linear unit (ReLU), sigmoid, and leaky ReLU and three different pooling functions, namely stochastic pooling, max pooling, and average pooling, for finding out the best model configuration. It was shown that the CNN model with leaky ReLU activation function and max pooling function gave the best results. Karasawa et al. [51] proposed a 3D-CNN based model for AD diagnosis using MR images. The architecture of proposed 3D-CNN is based on ResNet. It has 36 convolutional layers, a dropout layer, a pooling layer and a fully connected layer. Experiments conducted in the study demonstrated that the model outperformed several existing benchmarks.

Tang et al. [52] proposed an AD diagnosis model based on 3D Fine-tuning Convolutional Neural Network (3D-FCNN) using MR images. The authors demonstrated that the proposed model outperformed several existing benchmarks in terms of accuracy and robustness. Moreover, the authors compared the 3D-FCNN model with 2D-CNN and it was shown that the proposed model performed better than 2D-CNN in binary as well as multi-class classification. Spasov et al. [53] proposed a multi-modal framework based on CNN for AD diagnosis using structural MRI, genetic measures and clinical assessment. The devised framework had much fewer parameters as compared to the other CNN models such as VGGNet, AlexNet, etc. This made the framework faster and less susceptible to problems such as over-fitting in case of scarce-data scenarios.

Wang et al. [54] proposed a CNN based model for AD diagnosis using two crucial MRI modalities, namely fMRI and Diffusion Tensor Imaging (DTI). The model classified the subjects into three categories: AD, amnestic MCI and normal controls (NC). The authors proved that the proposed model performed better on multi-modal MRI than individual fMRI and DTI. Islam and Zhang [55] proposed a CNN-based model for AD diagnosis in the early stage using MR images. The authors trained the model using OASIS dataset, which is an imbalanced dataset. They used data augmentation to handle the imbalanced nature of the OASIS dataset. Experimental results demonstrated that the proposed model performed better than several state-of-the-art models. The authors plan to apply the proposed model to other AD datasets as well in future.

Yue et al. [56] proposed a CNN-based model for AD diagnosis using structural MR images. The model classified the subjects into four categories: AD, EMCI, LMCI and NC. Experiments carried out in the research work demonstrated that the proposed model outperformed several benchmarks. Jian et al. [57] proposed a transfer learning-based approach for AD diagnosis using structural MRI. VGGNet16 trained on the ImageNet dataset was used as a feature extractor for AD classification. The proposed approach successfully classified the input into three different categories: AD, MCI and CN. Huang et al. [58] designed a multi-modal model based on 3D-VGG16 for the diagnosis of AD using MRI and FDG-PET modalities. The study demonstrated that the model does not require segmentation of the input. Moreover, the authors showed that the hippocampus of the brain is a crucial Region of Interest (ROI) for AD diagnosis. The authors intend to include other modalities as well in future.

Goceri [59] proposed an approach based on 3D-CNN for AD diagnosis using MR Images. The proposed approach used Sobolev gradient as the optimizer, leaky ReLU as the activation function, and Max Pooling as the pooling function. The research work demonstrated that the combination of optimizer, activation function and pooling function implemented outperformed all the other combinations. Zhang et al. [60] utilized two independent CNNs for analyzing MR images and PET images separately. Then, correlation analysis of the outputs of the CNNs was performed to obtain the auxiliary diagnosis of AD. Finally, the auxiliary diagnosis result was combined with the clinical psychological diagnosis to obtain a comprehensive diagnostic output. The authors demonstrated that the proposed architecture is easy to implement and generates results closer to the clinical diagnosis.

Basheera and Ram [61] proposed a model based on CNN for AD diagnosis using MR images. The MR images were divided into voxels first. Gaussian filter was used to enhance the quality of voxels and a skull stripping algorithm was used to filter out irrelevant portions from the voxels. Independent component analysis was applied to segment the brain into different regions. Finally, segmented gray matter was fed as input to the proposed model. Experimental results demonstrated that the proposed model outperformed several state-of-the-art models. Spasov et al. [62] proposed a parameter-efficient CNN model for predicting the MCI to AD conversion using structural MRI, demographic data, neuropsychological data, and APOe4 genetic data. Experiments carried out in the research work demonstrated that the proposed model performed better than several existing benchmarks.

Ahmad and Pothuganti [63] performed a comparative analysis of SVM, Regional CNN (RCNN) and Fast Regional CNN for AD diagnosis. The study demonstrated that the Fast RCNN outperformed the other techniques. Lopez-Martin et al. [64] proposed a randomized 2D-CNN model for AD diagnosis in the early stage using MEG data. The research work demonstrated that the proposed model outperformed the classic machine learning techniques in AD diagnosis. Jiang et al. [65] proposed an eight-layered CNN model for AD diagnosis. The proposed model implemented batch normalization, data augmentation and drop-out regularization for achieving high accuracy. The authors compared the proposed model with several existing techniques, and it was demonstrated that the proposed model outperformed them.

Nawaz et al. [66] proposed a 2D-CNN based model for AD diagnosis using MRI data. The proposed model classified the input images into three groups: AD, MCI and NC. The authors compared the proposed model with AlexNet and VGGNet architectures, and it was demonstrated that the proposed model outperformed these architectures. Bae et al. [67] modified the Inception-v4 model pre-trained on ImageNet dataset for AD classification using MRI data. The study used datasets from subjects with two different ethnicities. The study demonstrated that the model has the potential to be used as a fast and accurate AD diagnostic tool. Jo et al. [68] proposed a model based on CNN for finding the correlation between tau deposition in the brain and probability of having AD. The study also identified the regions in the brain that are crucial for AD classification. According to the study, these regions include hippocampus, para-hippocampus, thalamus and fusiform.

Janghel and Rathore [69] proposed a VGG-16 based CNN model for AD diagnosis using fMRI and PET data. The CNN model carried out feature extraction and for classification, DT, SVM, KNN, and linear discriminate were used. It was observed that in the case of fMRI data, SVM, KNN, and linear discriminant classifiers achieved the best results. However, in the case of PET, KNN outperformed others. The authors also compared the proposed model with several existing studies, and it was demonstrated that the proposed model outperformed them. Sathiyamoorthi et al. [70] proposed a CNN-based model for AD diagnosis using MR images. The authors applied pre-processing techniques including 2D-ABF algorithm, AHA algorithm, AMS-MEM algorithm and 2D-GLCM for making it suitable for classification. The study demonstrated that the proposed model outperformed existing state-of-the-art techniques.

Mehmood et al. [71] proposed a CNN-based model called Siamese Convolutional Neural Network (SCNN) for the classification of dementia into different stages, namely Moderate Alzheimer’s Disease (MAD), Mild Dementia (MD), Very Mild Dementia (VMD), and No Dementia (ND). The authors demonstrated that even though the model was trained on a small dataset, it generated accurate results. They compared the proposed model with five state-of-the-art studies and it was demonstrated that the model outperformed them. Raju et al. [72] proposed a cascaded 3D-CNN for AD diagnosis using structural MR images. The proposed CNN model mined features from the input and SVM was used for the classification of the input based on the features extracted by the CNN model. The authors revealed that the model outperformed several benchmark models.

Sun et al. [73] modified the V-Net architecture for segmentation of bilateral hippocampi from 3D-MR images of the brain and for AD diagnosis. The authors demonstrated that the segmentation of hippocampi accurately is highly crucial for building an efficient AD diagnosis model. They analyzed the performance of the proposed architecture against several segmentation and classification models and it was reported that the model outperformed them. Dyrba et al. [74] built a CNN model for AD diagnosis using MR images. The authors assessed the co-relation between relevance score and volume of the hippocampus to check the utility of the approach in clinical settings. It was observed that hippocampal atrophy is the most crucial factor for AD detection.

Feng et al. [75] proposed a 3D-CNN-SVM model for AD diagnosis using MR images. The proposed model extracted features from the input MR images using 3D-CNN and classified the MR Images using SVM based on the features extracted by 3D-CNN. The study compared the performance of the proposed model with 2D-CNN and 3D-CNN, and it was demonstrated that 3D-CNN-SVM outperformed 2D-CNN and 3D-CNN. Solano-Rojas and Villalón-Fonseca [76] proposed a CNN based on DenseNet Bottleneck-Compressed architecture for AD diagnosis using MR images. The proposed model classified the input into five different categories, CN, EMCI, MCI, LMCI and AD, with an average accuracy of 86%.

Amini et al. [77] performed a comparative analysis RF, linear discrimination analysis (LDA), DT, SVM, KNN, and CNN for AD diagnosis using functional MRI data. It was observed that CNN outperformed all the other techniques and had the ability to model the AD severity efficiently. Turkson et al. [78] proposed a framework for AD classification based on MRI data consisting of two stages: discriminative AD features were extracted using an unsupervised Convolutional Spiking Neural Network and classification was performed using a supervised CNN. The study demonstrated that the proposed framework had an efficient discriminative capability for the diagnosis of AD. The authors intend to fuse clinical data with the neuroimaging data for building a more robust AD diagnosis model in future.

Lee et al. [79] designed a model based on CNN for brain age prediction in cognitively unimpaired individuals and AD patients. It was observed that in AD patients, the brain age gap was much larger as compared to the cognitively unimpaired individuals. Greve et al. [80] developed a tool using CNN for segmentation of sub-cortical limbic structures for early detection of AD. Ushizima et al. [81] designed a pipeline in order to extract tau associated features for AD classification using CNN.

### 3.3. AE for AD Diagnosis

The following studies utilized AE for AD diagnosis. Lu et al. [82] proposed a SAE-based model for predicting the progression of AD. The proposed model was named Multi-scale and Multi-modal Deep Neural Network (MMDNN) as it integrated information from multiple areas of the brain scanned using MRI and FDG-PET. Experiments carried out in the research work demonstrated that analyzing both MRI and FDG-PET gives better results than the single modal settings. Liu et al. [83] designed a SAE-based model for the diagnosis of AD in its early stage. The authors demonstrated that the designed model performed well even in the case of limited training data. Moreover, the authors analyzed the performance of the model against Single-Kernel SVM and Multi-Kernel SVM, and it was revealed that the proposed model outperformed these models.

Lu et al. [84] proposed a DL model based on SAE for discriminating pre-symptomatic AD and non-progressive AD in subjects with MCI using metabolic features captured with FDG-PET. The parameters in the model were initialized using greedy layer-wise pre-training. Softmax-layer was added for performing the classification. The proposed model was compared with the existing benchmark techniques that utilized FDG-PET for capturing the metabolic features, and it was shown that it performed better than those techniques.

### 3.4. RNN for AD Diagnosis

Lee et al. [85] proposed a RNN-based model that extracted temporal features from multi-modal data for forecasting the conversion of MCI subjects to AD patients. The data were fused between different modalities, including demographic information, MRI, CSF biomarkers and cognitive performance. The authors proved that the model outperformed the existing benchmarks. Furthermore, it was shown that the multi-modal model outperformed the individual single-modal models.

### 3.5. DBN for AD Diagnosis

Ortiz et al. [86] proposed two methods based on DBN for the early diagnosis of AD. These methods worked on fused functional and structural MRI scans. The first one, named as DBN-voter, consisted of an ensemble of DBN classifiers and a voter. Four different voting schemes were analyzed in the study, namely majority voting, weighted voting, classifiers fusion using SVM, and classifiers fusion using DBN. As the second model, FEDBN-SVM used DBNs as feature extractors and carried out classification using SVM. It was demonstrated that FEDBN-SVM outperformed DBN-voter in addition to the existing benchmarks, and in the case of DBN-voter, DBNs with classifiers fusion using SVM performed better.

### 3.6. GAN for AD Diagnosis

Ma et al. [87] proposed a GAN-based model for the differential diagnosis of frontotemporal dementia and AD pathology. The model extracted multiple features from MR images for classification. Moreover, data augmentation was performed in order to avoid over-fitting caused due to limited data problem. Experimental analysis carried out in the research work revealed that the model showed promising results in the differential diagnosis of frontotemporal dementia and AD pathology. The authors claimed that the proposed model could be used for the differential diagnosis in other neurodegenerative diseases as well.

### 3.7. Hybrid DL Models for AD Diagnosis

The following studies utilized hybrid DL models for AD diagnosis. Zhang et al. [88] proposed 3D Explainable Residual Self-Attention Convolutional Neural Network (3D ResAttNet) for diagnosis of AD using structural MR images. The proposed model is a CNN with a self-attention residual mechanism, and explainable gradient-based localization class activation mapping was employed that provided visual analysis of AD predictions. The self-attention mechanism modeled the long-term dependencies in the input and the residual mechanism dealt with the vanishing gradient problem. The authors compared the proposed model with 3D-VGGNet and 3D-ResNet, and it was shown that the proposed model performed better than these models.

Payan and Montana [89] formulated a model based on 3D-CNN for the prediction of AD using MR images. The study employs Sparse AE for pre-training the convolutional filters. Experiments conducted in the research work revealed that the model outperformed the existing benchmarks. Hosseini et al. [90] proposed a hybrid model consisting of AE and 3D-CNN for early stage diagnosis of AD. The variations in anatomical shapes of brain images were captured by AE, and classification was carried out using 3D-CNN. The authors compared the proposed model with the existing benchmarks, and it was established that the proposed model outperformed those techniques. Moreover, the authors plan to apply the proposed model for the diagnosis of other conditions such as autism, heart failure and lung cancer.

Vu et al. [91] proposed an AD detection system based on High-Level Layer Concatenation Auto-Encoder (HiLCAE) and 3D-VGG16. HiLCAE was used as a pre-trained network for initializing the weights of 3D-VGG16. The proposed system worked on the fused MR and PET images. Experiments carried out in the research work demonstrated that the proposed system detected AD with good accuracy. The authors intend to develop deeper networks for both HiLCAE and VGG16 in future so as to improve the accuracy further.

Warnita et al. [92] proposed a gated CNN-based approach for AD diagnosis using speech transcripts. The proposed approach captured temporal features from speech data and performed classification based on the extracted features. The authors plan to apply the proposed approach to different languages in the future. Feng et al. [93] proposed a hybrid model consisting of Stacked Bidirectional RNN (SBi-RNN) and two 3D-CNNs for diagnosis of AD in its early stage. CNNs extracted preliminary features from MRI and PET images, while SBi-RNN abstracted discriminative features from the cascaded output of CNNs. The output from SBi RNN was fed to a softmax classifier that generated the model output. Experiments conducted in the study demonstrated that the proposed model outperformed state-of-the-art models.

Li and Liu [94] proposed a framework consisting of Bidirectional Gated Recurrent Unit (BGRU) and DenseNets for hippocampus analysis-based AD diagnosis. The DenseNets were trained to capture the shape and intensity of MR images and BGRU abstracted high-level features between the right and left hippocampus. Finally, a fully connected layer performed classification based on the extracted features. Experiments conducted in the study revealed that the proposed framework generated promising results. Oh et al. [95] proposed a model based on end-to-end learning using CNN for carrying out the following classifications: AD versus NC, pMCI (probable MCI) versus NC, sMCI (stable MCI) versus NC, and pMCI versus sMCI. The authors utilized Convolutional Auto-Encoder for performing AD versus NC classification, and transfer learning was implemented to perform pMCI versus sMCI classification. Experiments carried out in the study showed that the proposed model worked better than several existing benchmarks.

Chien et al. [96] developed a system for assessing the risk of AD based on speech transcripts. The system consisted of three components: a data collection component that fetched data from the subject, a feature sequence generator that converted the speech transcripts into the features, and an AD assessment engine that determined whether the person had AD or not. The feature sequence generator was built using a deep convolutional RNN, and the AD assessment engine was realized using a bidirectional RNN with the gated recurrent unit. Experimental analysis carried out in the research work revealed that the system gives promising results.

Kruthika et al. [97] proposed a hybrid model consisting of 3D Sparse AE, CNN and capsule network for detection of AD in its early stage. The authors revealed that the hybrid model worked better than the 3D-CNN. Basher et al. [98] proposed an amalgam of Hough CNN, Discrete Volume Estimation-CNN (DVE-CNN) and DNN for AD diagnosis using structural MR images. Hough CNN has been used to localize right and left hippocampi. DVE-CNN was utilized to mine volumetric features from the pre-processed 2D patches. Finally, DNN classified the input based on the features extracted using DVE-CNN. The study demonstrated that the proposed approach outperformed the existing benchmarks by a good margin.

Roshanzamir et al. [99] utilized a bidirectional encoder with logistic regression for early prediction of AD using speech transcripts. The authors implemented the concept of data augmentation for dealing with the limited dataset problem. Experiments conducted in the study demonstrated that the bidirectional encoder with logistic regression outperformed the existing benchmarks. Zhang et al. [100] proposed a densely connected CNN with attention mechanism for AD diagnosis using structural MR images. The densely connected CNN extracted multiple features from the input data, and the attention mechanism fused the features from different layers to transform them into complex features based on which final classification was performed. It was established that the model outperformed several existing benchmark models.

**Table 1:** Summary of research works.

Work	Year	Biomarker	DL Method	Dataset	Performance
[83]	2014	MRI and PET	SAE	ADNI-311 subjects (AD-65, cMCI-67, ncMCI-102, NC-77)	Accuracy (NC/AD): 87.76% Accuracy (NC/MCI): 76.92%
[88]	2015	MRI	Residual Self Attention 3D Convolutional Neural Network	ADNI-835 subjects (AD-200, MCI-404, NC-231)	Accuracy (NC/AD): 91.3% ± 0.012 Accuracy (sMCI/pMCI): 82.1% ± 0.092
[89]	2015	MRI	CNN + Sparse AE	ADNI-2265 subjects (AD-755, MCI-755, HC-755)	Accuracy (HC/MCI/AD): 89.47% Accuracy (HC/AD): 95.39% Accuracy (AD/MCI): 86.84% Accuracy (HC/MCI): 92.11%
[43]	2016	MRI	CNN	ADNI-805 subjects (AD-186, MCI-393, NC-226)	Accuracy (NC/ADI): 91.02% ± 4.29 Accuracy (NC/MCI): 73.02% ± 6.44 Accuracy (sMCI/pMCI): 74.82% ± 6.80
[44]	2016	MRI	CNN	ADNI-900 subjects (AD-300, MCI-300, HC-300)	Accuracy (HC/MCI/AD): 91.85%
[45]	2016	fMRI	CNN	ADNI-43 subjects (AD-28, NC-15)	Accuracy(NC/AD): 96.85%
[86]	2016	MRI and fMRI	DBN	ADNI-275 subjects (AD-70, MCI-111, LMCI-26, NC-68)	Accuracy (NC/AD): 90% Accuracy (MCI/AD): 84% Accuracy (NC/MCI): 83%
[90]	2016	MRI	CNN + AE	ADNI-210 subjects (AD-70, MCI-70, NC-70)	Accuracy (NC/MCI/AD): 89.1%
[46]	2016	MRI	CNN	ADNI-302 subjects (AD-211, HC-91)	Accuracy (HC/AD): 98.84%
[47]	2016	MRI and fMRI	CNN	ADNI (fMRI-144 subjects: AD-52, CN-92) ADNI(MRI-302 subjects: AD-211, CN-91)	Accuracy (fMRI (CN/AD)): 99.9% Accuracy (MRI (CN/AD)): 98.84%
[31]	2017	MRI	DNN	ADNI-240 subjects (AD-60, cMCI-60, MCI-60,HC-60)	Accuracy (HC/MCI/cMCI/AD): 53.7 ± 1.9%
[48]	2017	MRI	CNN	ADNI-504 subjects (AD-101, MCI-234, CN-169)	Accuracy (CN/MCI/AD): 96%
[84]	2018	MRI and FDG-PET	SAE	ADNI-1051 subjects (NC-304, sMCI-409, pMCI-112, AD-226)	Accuracy (NC/AD): 93.58%, Accuracy (sMCI/pMCI): 81.55%
[32]	2018	EEG	DNN	Data collected from Chosun University Hospital and Gwangju Optimal Dementia Center located in South Korea-20 subjects (MCI-10, HC-10)	Accuracy (NC/MCI): 59.3%
[82]	2018	MRI and FDG-PET images	SAE	ADNI-1242 subjects (sNC-360, sMCI-409, pNC: 18, pMCI-217, sAD-238)	Accuracy (sMCI/pMCI): 82.93%
[49]	2018	MRI	CNN	ADNI-1409 subjects (AD-294, MCI-763, HC-352), Milan dataset-229 subjects (AD-124, MCI-50, HC-55)	Accuracy (HC/AD): 98.2% Accuracy (HC/cMCI): 87.7% Accuracy (HC/sMCI): 76.4% Accuracy (cMCI/AD): 75.8% Accuracy (sMCI/AD): 86.3% Accuracy (cMCI/sMCI): 74.9%
[33]	2018	MRI and AV-45 PET data	DNN	ADNI-896 subjects (CN-248, AD-149, EMCI-296, LMCI-193)	Accuracy (CN/EMCI): 84% Accuracy (CN/LMCI): 84.1% Accuracy (CN/AD): 96.8% Accuracy (EMCI/LMCI): 69.5% Accuracy (EMCI/AD): 90.3% Accuracy (LMCI/AD): 80.2%
[34]	2018	EEG	DNN	Data collected from Medical Universities of Graz, Innsbruck and Vienna, as well as Linz General Hospital—188 subjects (Probable AD-133, Possible AD-55)	Mean Squared Error (Probable AD/Possible AD): 12.17
[91]	2018	MRI and FDG-PET	AE + CNN	ADNI-615 subjects (AD-193, MCI-215, NC-207)	Accuracy (MCI/AD): 93% Accuracy (NC/MCI): 95% Accuracy (NC/AD): 98.8% Accuracy (NC/MCI/AD): 91.13%
[50]	2018	MRI	CNN	OASIS dataset-126 subjects (AD-28, HC-98) and data from local hospitals-70 subjects (AD-70)	Accuracy (HC/AD): 97.65%
[51]	2018	MRI	CNN	ADNI-1728 subjects (AD-346, MCI-450, LMCI-358, NC-574)	Accuracy (NC/AD): 94% Accuracy (NC/MCI): 90% Accuracy (NC/MCI/AD): 87%
[52]	2018	MRI	CNN	ADNI-391 subjects (AD-150, MCI-129, NC-112)	Accuracy (NC/AD): 96.81% Accuracy (MCI/AD): 88.43% Accuracy (NC/MCI): 92.62% Accuracy (NC/MCI/AD): 91.32%
[35]	2018	Speech transcripts	DNN	DementiaBank dataset	AUC (MCI/AD): 0.815
[53]	2018	MRI, clinical assessment and genetic (APOe4) measures	CNN	ADNI-800 subjects (AD-200, MCI-400, NC-200)	Accuracy (NC/MCI/AD): 99%
[54]	2018	fMRI and Diffusion Tensor Imaging (DTI)	CNN	ADNI-105 subjects (AD-35, aMCI-30, NC-40)	Accuracy (NC/aMCI/AD): 92.06%
[92]	2018	Speech transcripts	Gated CNN	DementiaBank dataset-267 subjects (AD-169, HC-98)	Accuracy (HC/AD): 73.6%
[36]	2018	MRI and single nucleotide polymorphism (SNP) data	DNN	ADNI-721 subjects (AD-138, MCI-358, CN-225)	AUC (CN/MCI/AD): 0.992
[55]	2018	MRI	CNN	OASIS dataset-416 subjects	Accuracy (Non Demented/very Mild/Mild/Moderate): 93%
[93]	2018	MRI and PET	CNN + RNN	ADNI-397 subjects (AD-93, pMCI-76, sMCI-128, CN-100)	Accuracy (NC/AD): 94.29% Accuracy (NC/pMCI): 84.66% Accuracy (NC/sMCI): 64.47%
[56]	2018	MRI	CNN	ADNI-1663 subjects (AD-336, MCI-542, CN-785)	Accuracy (NC/LMCI): 94.5% Accuracy (NC/AD): 96.9% Accuracy (LMCI/AD): 97.2% Accuracy (EMCI/AD): 97.81% Accuracy (EMCI/LMCI): 94.8%
[37]	2019	gene expression and DNA methylation profiles	DNN	GSE33000 and GSE44770 (gene expression), prefrontal cortex GSE80970 (DNA methylation)	Accuracy (NC/AD): 82.3%
[38]	2019	MRI	DNN	OASIS-416 subjects	Accuracy (NC/AD): 86.66%
[57]	2019	MRI	CNN	ADNI-150 subjects (AD-50, CN-50, MCI-50)	Accuracy (CN/AD): 99.14% Accuracy (AD/MCI): 99.3% Accuracy (CN/MCI): 99.2%
[39]	2019	MRI	DNN	ADNI-291 subjects (AD-97, CN-194)	Accuracy (CN/AD): 67%
[94]	2019	MRI	CNN + RNN	ADNI-807 subjects (AD-194, MCI-397, NC-216)	Accuracy (NC/AD): 91.0% Accuracy (NC/MCI): 75.8% Accuracy (sMCI/pMCI): 74.6%
[95]	2019	MRI	AE+ CNN	ADNI-694 subjects (AD-198, NC-230, sMCI-101, pMCI-166)	Accuracy (AD/NC): 86.60% ± 3.66% Accuracy (pMCI/NC): 77.37% ± 3.55% Accuracy (sMCI/NC): 63.04% ± 4.16% Accuracy (pMCI/AD): 60.97% ± 5.33% Accuracy (sMCI/AD): 75.06% ± 3.86
[58]	2019	MRI and FDG-PET	CNN	ADNI-2145 subjects (AD-647, sMCI-441, pMCI-326, HC-731)	Accuracy (NC/AD): 90.10% Accuracy (NC/pMCI): 87.46% Accuracy (sMCI/pMCI): 76.90%
[59]	2019	MRI	CNN	ADNI-315 subjects (AD-185, HC-130)	Accuracy (HC/AD): 98.06%
[85]	2019	Demographic information, neuro-imaging phenotypes measured by MRI, cognitive performance, and CSF measurements	RNN	ADNI-1618 subjects (AD-338, MCI-865, CN-415)	Accuracy (CN/MCI/AD): 81%
[96]	2019	Speech transcripts	CNN + RNN	DementiaBank dataset	AUC (NC/AD): 0.838
[97]	2019	MRI	CNN + AE	ADNI-1941 subjects (AD-345, MCI-991, NC-605)	Accuracy (MCI/AD): 94.6% Accuracy (NC/AD): 92.98% Accuracy (NC/MCI): 94.04%
[60]	2019	MRI and PET	CNN	ADNI-392 subjects (AD-91, MCI-200, CN-101)	Accuracy (NC/AD): 98.47% Accuracy (NC/MCI): 85.74% Accuracy (AD/MCI): 88.20%
[61]	2019	MRI	CNN	ADNI-1820 images (AD-635, MCI: 548, CN: 637)	Accuracy (CN/MCI/AD): 86.9% Accuracy (CN/AD): 100% Accuracy (MCI/AD): 96.2% Accuracy (CN/MCI): 98%
[40]	2019	MRI	DNN	ADNI-1737 subjects	AUC (NC/MCI/AD): 0.866
[62]	2019	MRI and clinical features	CNN	ADNI-785 subjects (AD-192, MCI-409, HC-184)	Accuracy (MCI/AD): 86%
[87]	2020	MRI	GAN	ADNI-1114 subjects and Frontotemporal Lobar Degeneration Neuroimaging Initiative (NIFD)-840 subjects	Accuracy (NC/AD): 88.28%
[63]	2020	MRI	CNN	ADNI	Test time (NC/AD): 0.2 s
[64]	2020	MEG	CNN	Data collected from Centre for Biomedical Technology, Spain-132 subjects (MCI-78, HC-54)	F1-Score (HC/MCI) = 0.92
[65]	2020	MRI	CNN	OASIS dataset-126 subjects (AD-28, HC-98) and data from local hospitals-70 subjects (AD-70)	Accuracy (HC/AD): 97.76% ± 0.41
[66]	2020	MRI	CNN	ADNI-159 subjects (AD-45, MCI-62, NC-52)	Accuracy (NC/MCI/AD): 99.89%
[67]	2020	MRI	CNN	ADNI-390 subjects (AD-195, CN-195), SNUBH-390 subjects (AD-195, CN-195)	Accuracy (ADNI (CN/AD)): 89% Accuracy (SNUBH (CN/AD)): 88%
[69]	2020	fMRI and PET	CNN	fMRI ADNI dataset-54 subjects (AD-27, HC-27) PET ADNI dataset-2675 images (AD-900, HC-1775)	Accuracy (fMRI dataset (HC/AD)): 99.95% Accuracy (PET ADNI (HC/AD)): 73.46%
[70]	2020	MRI	CNN	Kaggle’s MRI dataset	Accuracy (MCI/AD): 96%
[41]	2020	MRI	DNN	ADNI-819 subjects (AD-192, MCI-398, CN-229) and NIMHANS-99 (AD-39, CN-60)	Accuracy (ADNI (CN/MCI/AD)): 99.50% Accuracy (NIMHANS (CN/AD)): 98.40%
[71]	2020	MRI	CNN	OASIS-382 images (No Dementia: 167, Very Mild Dementia-87, Mild Dementia-105, Moderate AD-23)	Accuracy (No Dementia/Very Mild Dementia/Mild Dementia/Moderate AD): 99.05%
[72]	2020	MRI	CNN	ADNI-465 subjects (AD-132, MCI-181, CN-152)	Accuracy (CN/MCI/AD): 97.77%
[73]	2020	MRI	CNN	ADNI-132 subjects (AD-25, MCI-61, CN-46)	Accuracy (CN/MCI/AD):84%
[74]	2020	MRI	CNN	ADNI-GO/2-663 subjects ADNI-3-575 subjects AIBL-606 subjects DELCODE-474 subjects	Accuracy (ADNI-GO/2): 86.25% Accuracy (ADNI-3): 74.375% Accuracy (AIBL): 79.225% Accuracy (DELCODE): 78%
[75]	2020	MRI	CNN	ADNI-469 subjects (AD-153, MCI-157, CN-159)	Accuracy (NC/MCI/AD): 92.11% ± 2.31 Accuracy (NC/AD): 99.10% ± 1.13 Accuracy (NC/MCI): 98.90% ± 2.78 Accuracy (MCI/AD): 89.40% ± 6.90
[68]	2020	Tau-PET	CNN	ADNI-300 subjects (AD-66. EMCI-97, LMCI-71, CN-66)	Accuracy (CN/AD): 90.8%
[98]	2021	MRI	CNN + DNN	Gwangju Alzheimer’s and Related Dementia (GARD)	Accuracy (NC/AD): 94.02%
[99]	2021	Speech transcripts	Bidirectional encoder with logistic regression	DementiaBank dataset-269 subjects (AD-170, HC-99)	Accuracy (HC/AD): 88.08%
[100]	2021	MRI	CNN with attention mechanism	ADNI-968 subjects (AD-280, cMCI-162, ncMCI-251, NC-275)	Accuracy (NC/AD): 97.35% Accuracy (NC/MCI): 87.82% Accuracy (MCI/AD): 78.79%
[76]	2021	MRI and PET	CNN	ADNI-5556 images (AD-718, EMCI-1222, MCI-1274, LMCI-636, SMC-186, CN-1520)	Accuracy (CN/EMCI/MCI/LMCI/AD): 86%
[77]	2021	fMRI	CNN	ADNI-675 subjects	Accuracy (Low AD): 98.1% Accuracy (Mild AD): 95.2% Accuracy (Moderate AD): 89% Accuracy (Severe AD): 87.5%
[78]	2021	MRI	CNN	ADNI-450 subjects (AD-150, MCI-150, NC-150)	Accuracy (NC/AD): 90.15% ± 1.1 Accuracy (MCI/AD): 87.30% ± 1.4 Accuracy (NC/MCI): 83.90% ± 2.5
[42]	2021	Genetic Measures	DNN	MCSA-266 subjects	*p*-value ˂ 1 × $10^{-3}$
[79]	2021	FDG-PET	CNN	MCSA	Mean Absolute Error: 2.8942
[80]	2021	MRI	CNN	HABS	Error rate < 1%
[81]	2021	Tau-PET and MRI	CNN	Tau-PET and MRI images from two human brains	Area under Curve: 0.88

## 4. Discussion

Effective and precise identification of AD is critical for the start of productive therapy. Early detection of AD, in particular, is critical for the therapeutic improvement and, ultimately, for optimal patient care. We conducted a systematic assessment of the diagnostic classification of AD based on DL models. We looked at seventy research articles from 2014 to 2021 and categorized them based on DL technique, type of biomarker, dataset and performance metric (Table 1).

**Figure 4:**
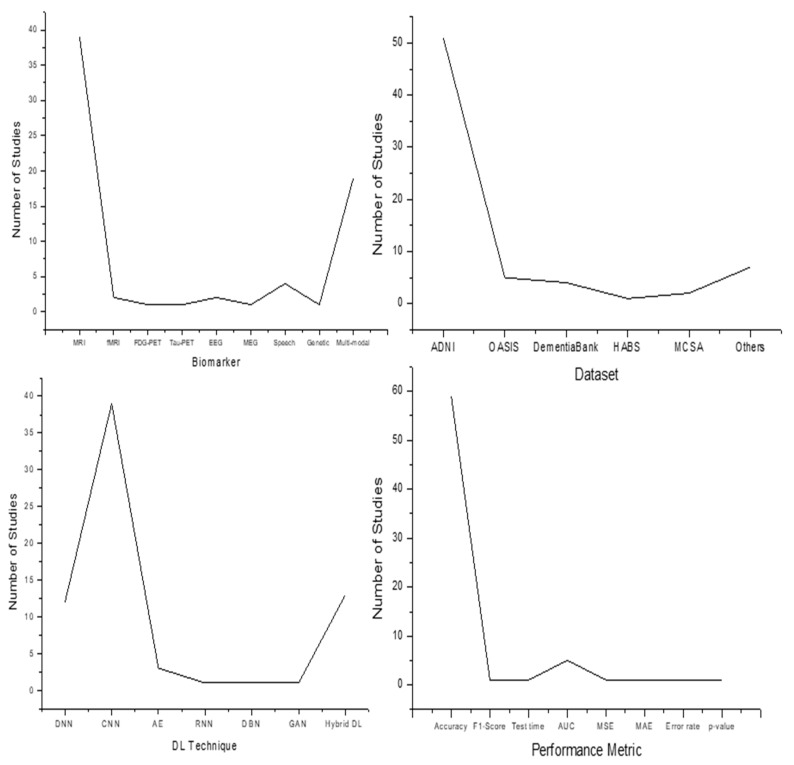
Number of studies versus biomarker, datasets, DL technique and performance metric.

Out of seventy studies, thirty-nine utilized CNN, twelve utilized DNN, three utilized AE, one utilized RNN, one utilized DBN, one utilized GAN and thirteen utilized hybrid DL models. The majority of the studies utilized CNN for AD diagnosis, followed by hybrid DL models. The accuracies of studies ranged between 53.17% and 99.95%. With regards to biomarkers, thirty-nine utilized MRI scans, two utilized fMRI scans, one utilized FDG-PET, one utilized Tau-PET, two utilized EEG, one utilized MEG, four utilized speech transcripts, one utilized genetic measures, and nineteen utilized multi-modal biomarkers. The maximum number of studies utilized MRI followed by multi-modal biomarkers. Regarding datasets, fifty-one studies utilized the ADNI dataset, five utilized the OASIS dataset, DementiaBank was used by four, HABS by one, MCSA by two, and seven studies utilized other datasets for AD diagnosis. The majority of studies utilized the ADNI dataset for AD diagnosis. Regarding performance metrics, fifty-nine studies utilized accuracy, five utilized AUC, one study utilized F1-score, one utilized test time, one utilized error rate, one utilized MAE, one utilized *p*-value, and one utilized MSE as the performance metric. These statistics are shown in Figure 4. The following summarizes the key findings from the literature review:

- DL techniques outperform conventional machine learning techniques in AD diagnosis.
- DNN outperforms the shallow neural network architectures in AD diagnosis.
- Conventional machine learning techniques such as Random Forest, KNN, SVM can be used to assist DL models in feature selection and discrimination processes.
- Multimodal classification models outperform single-modal settings.
- Fusion of typical neurophysiological data with MRI and PET enhances the efficiency of the AD classification models.
- Bayesian method and greedy layer-wise pre-training are effective techniques for initializing the DL model parameters such as learning rate, drop-out rate, number of hidden layers, and number of nodes in each layer.
- Due to the shift-invariant and scale-invariant properties, CNN has got a massive scope in medical image analysis.
- Transfer learning and data augmentation are suitable for avoiding over-fitting in DL models.
- CNN model with leaky ReLU activation function and Max pooling function gives the best results as compared to other combinations of activation functions and pooling functions.
- Models built on multi-modal MRI (fMRI and DTI) perform better than models built on individual fMRI and DTI.
- Hippocampus of the brain is a crucial ROI for AD diagnosis, and hippocampal atrophy is the most crucial factor for AD diagnosis.
- Batch normalization, data augmentation and drop-out regularization generate efficient AD classification models.
- Selecting the appropriate pre-processing and segmentation techniques are crucial for building efficient DL models for AD diagnosis.
- Unsupervised DL techniques such as auto-encoders are effective for limited data scenarios.
- Hybrid DL models perform better than individual DL models.

## 5. Challenges and Future Research Directions

Despite the significant advancements made by DL techniques in the diagnosis of AD, there are still some critical hurdles that need to be overcome. These challenges are as follows:

- Over-fitting: DL algorithms are multilayered algorithms that need a lot of processing power and have millions of parameters. Convergence of these algorithms necessitates a huge quantity of data in proportion to the number of parameters. Although there are no hard and fast rules about how much data are needed to train DL algorithms, empirical research suggests that ten times more training data are needed than the number of parameters. Given the widespread availability of images, text and videos on the internet, it is no wonder that disciplines such as computer vision and natural language processing have experienced the fastest advancements due to DL. Neuro-imaging data, on the other hand, is largely decentralized and housed locally within hospital systems, with privacy restrictions that make it difficult to access for research. Furthermore, due to the complexity of disease processes and patient presentations, obtaining solid ground truth labels for neurological diseases including AD is exceedingly costly, and requires expert knowledge. The scarcity of labeled data continues to be a major stumbling block in the advancement of DL in AD diagnosis.

Over-fitting is always a possibility when training a complicated classifier on a limited dataset. DL models have a strong tendency to fit data well, but this does not imply that they generalize well. Many studies have employed various tactics to mitigate over-fitting, such as regularization, early stopping, and drop-out. While the algorithm’s performance on a separate test data set can be used to assess over-fitting, the algorithm may not work well on similar images obtained in other facilities, on different scanners, or with patients with different demographics. Larger datasets from multiple locations are often gathered in diverse ways, with marginally varied image attributes, using different scanners and protocols, resulting in poor performance. Moreover, it has been observed that without consistent criteria, data augmentation will not be able to adequately address difficulties with limited datasets. Overcoming this issue is a crucial topic of study.

- Data Quality: DL algorithms are intrinsically unsuited to healthcare data in general. Electronic medical records are made up of highly heterogeneous clinical notes, a jumble of diverse codes, and other patient details often containing missing and incomplete data. This intrinsic complication of healthcare data makes it impractical for DL algorithms to separate signal from noise.
- Interpretability and Transparency: Expert intervention in preprocessing procedures for feature selection and extraction from images in traditional machine learning algorithms may be required. DL, on the other hand, does not require human mediation and digs out features straight from the input data, therefore data preprocessing is not usually required. This enables greater flexibility in feature extraction based on a variety of inputs. As a result, DL can produce an effective model at each time of the run. Because of this flexibility, DL has outperformed conventional machine learning methods that rely on preprocessing. However, this element of DL inherently introduces uncertainty about which features will be mined at each epoch, and it is hard to explain which individual features were extracted from the network unless there is a dedicated design for the feature. It is also hard to figure out how those selected characteristics lead to a conclusion and the relative relevance of various features or subclasses of features due to the intricacy of the DL algorithms, which consists of several hidden layers. This is a significant restriction for AD research in which it is desirable to understand the importance of specific traits in order to create models. These intricacies and uncertainties tend to obscure the process of attaining high accuracy, making it more difficult to rectify any biases in the dataset.
- Reproducibility: The performance of DL algorithms is affected by the values of hyper-parameters such as learning rate, drop-out, number of epochs, batch size, momentum, etc. It is crucial to use the same choice of hyper-parameters on numerous levels to get the same experimental result. Even if hyper-parameters and random seeds are not offered in most circumstances, it is necessary to keep the same code bases. The randomization of the training technique and the ambiguity of the setup may make it impossible to replicate the study and acquire the same findings.

## 6. Conclusions

In developed countries, AD is a prominent cause of death. Although the premature diagnosis of AD is a critical and strenuous task, the employment of computer-based methods in conjunction with medical specialists can provide an effective diagnostic approach. In recent years, it has been observed that DL techniques have the capability to model the progression of AD efficiently and accurately based on neuroimaging data and hence can contribute to slowing down its advancement. Motivated to unfold the potential of DL techniques in AD diagnosis, this study reviewed the current benchmarks in the arena. An in-depth literature review of AD diagnosis using DL techniques, such as feed-forward DNN, CNN, AE, RNN, DBN, GAN and hybrid DL models, was carried out. The study also explored the different biomarkers and datasets for AD diagnosis. The biomarkers that are crucial for AD diagnosis include MRI, fMRI, FDG-PET, amyloid-PET, Tau-PET, EEG, MEG, speech transcripts, genetic measures, and CSF measures. The datasets that are available for AD diagnosis are ADNI dataset, OASIS dataset, DementiaBank dataset, HABS dataset, and MCSA dataset. A systematic assessment of the literature was carried out, and the trends and key findings were discussed. It was observed that CNN is the most widely used DL technique for AD diagnosis, followed by hybrid DL models. In the case of biomarkers and datasets, MRI and ADNI have been extensively used in the AD diagnosis studies using DL, respectively. However, it was discerned that regardless of the fact that DL has made massive advancements in AD diagnosis, there are still some impediments that need to be addressed. These impediments include over-fitting, data quality, interpretability, transparency, and reproducibility.

## Data Availability

Not applicable.

## Abbreviation

ABF	Adaptive Bilateral Filter
AD	Alzheimer’s Disease
ADNI	Alzheimer’s Disease Neuroimaging Initiative
AE	Auto-Encoder
AHA	Adaptive Histogram Adjustment
AMS-MEM	Adaptive Mean Shift Modified Expectation Maximization
AUC	Area under Curve
BBB	Blood-Brain Barrier
BDAE	Boston Diagnostic Aphasia Examination
BGRU	Bidirectional Gated Recurrent Unit
BPTT	Back-Propagation Through Time
CN	Cognitively Normal
CNN	Convolutional Neural Network
CSF	Cerebrospinal Fluid
DBN	Deep Belief Network
DL	Deep Learning
DNA	Deoxyribonucleic Acid
DNN	Deep Neural Network
DT	Decision Tree
DTI	Diffusion Tensor Imaging
DVE	Discrete Volume Estimation
EEG	Electroencephalography
EMCI	Early Mild Cognitive Impairment
FCNN	Fine tuning Convolutional Neural Network
FDG-PET	Fluorodeoxyglucose-Positron Emission Tomography
fMRI	Functional Magnetic Resonance Imaging
GAN	Generative Adversarial Network
GLCM	Gray Level Co-Occurrence Matrix
HABS	Harvard Aging Brain Study
HC	Healthy Control
HiLCAE	High-Level Layer Concatenation Auto-Encoder
HOG	Histogram of Oriented Gradients
KNN	K Nearest Neighbour
LDA	Linear Discriminant Analysis
LMCI	Late Mild Cognitive Impairment
LR	Logistic Regression
MAD	Moderate Alzheimer’s Disease
MCI	Mild Cognitive Impairment
MCSA	Mayo Clinic Study of Aging
MEG	Magnetoencephalography
MMDNN	Multi-scale and Multi-modal Deep Neural Network
MR	Magnetic Resonance
MRI	Magnetic Resonance Imaging
MSE	Mean Squared Error
NC	Normal Control
ND	No Dementia
OASIS	Open Access Series of Imaging Studies
PET	Positron Emission Tomography
pMCI	probable Mild Cognitive Impairment
PUP	PET Unified Pipeline
RCNN	Regional Convolutional Neural Network
RF	Random Forest
RNN	Recurrent Neural Network
ROI	Region of Interest
SAE	Stacked Auto- Encoder
SBi-RNN	Stacked Bidirectional RNN
SCNN	Siamese Convolutional Neural Network
sMCI	stable Mild Cognitive Impairment
SVM	Support Vector Machine
VMD	Very Mild Dementia
